# Correction: The inactivation mechanism of chemical disinfection against SARS-CoV-2: from MD and DFT perspectives

**DOI:** 10.1039/d0ra90127j

**Published:** 2021-01-15

**Authors:** Chunjian Tan, Chenshan Gao, Quan Zhou, Willem Van Driel, Huaiyu Ye, Guoqi Zhang

**Affiliations:** Electronic Components, Technology and Materials, Delft University of Technology 2628 CD Delft The Netherlands G.Q.Zhang@tudelft.nl; School of Microelectronics, Southern University of Science and Technology Shenzhen 518055 China yehy@sustech.edu.cn; The Key Laboratory of Optoelectronic Technology & Systems, Education Ministry of China, College of Optoelectronic Engineering, Chongqing University Chongqing 400044 China; Shenzhen Institute of Wide-Bandgap Semiconductors No. 1088, Xueyuan Rd, Xili, Nanshan District Shenzhen Guangdong China; Engineering Research Center of Integrated Circuits for Next-Generation Communications, Ministry of Education Nanshan District Shenzhen Guangdong China; Signify High Tech Campus 7 5656AE Eindhoven The Netherlands Willem.van.driel@signify.com

## Abstract

Correction for ‘The inactivation mechanism of chemical disinfection against SARS-CoV-2: from MD and DFT perspectives’ by Chunjian Tan *et al.*, *RSC Adv.*, 2020, **10**, 40480–40488, DOI: 10.1039/D0RA06730J.

The authors regret that one of the affiliations (affiliation f) was incorrectly omitted in the original manuscript. The corrected list of affiliations is as shown below.

The Royal Society of Chemistry apologises for these errors and any consequent inconvenience to authors and readers.

## Supplementary Material

